# Anatomical Landmarks for Safe Elevation of the Deep Inferior Epigastric Perforator Flap: A Cadaveric Study

**Published:** 2010-05-28

**Authors:** Saeed Chowdhry, Ron Hazani, Philip Collis, Bradon J. Wilhelmi

**Affiliations:** University of Louisville, Louisville, KY 40241

## Abstract

**Background:** Breast reconstruction techniques have focused increasingly on using autologous tissue, with emphasis being placed on employing muscle sparing adipocutaneous flaps to reduce abdominal wall complications such as hernias, bulges, weakness, and length of hospital stay. The result has been the emergence of the deep inferior epigastric perforator (DIEP) flap for breast reconstruction. Isolating perforator vessels challenges most surgeons. We describe surface anatomical landmarks to predict the location of the deep inferior epigastric artery (DIEA) and its perforators to aid in the efficient elevation of this flap. **Methods:** Ten fresh hemi-abdomens were dissected with loupe magnification. The DIEA and its perforators were identified, and measurements in relation to the rectus muscle, xiphoid, umbilicus, and pubis were taken. Statistical analysis was undertaken to determine distance ratios to account for variance in patient size. **Results:** Average distance from the xiphoid to umbilicus was 18.2 ± 1.27 cm. The distance from the umbilicus to pubis was 14.9 ± 2.3 cm. The vertical distance from the umbilicus to the DRJ (DIEA rtctus junction) was 10.45 ± 1.58 cm, and the vertical distance from the level of the umbilicus to where the first DIEA perforator traverses the RAM was 7.4 ± 1.64 cm. The distance between the umbilicus and the DRJ is approximately 0.7 times the distance between the umbilicus and the pubic symphysis. The distance between the umbilicus and the first perforator is approximately 0.5 times the distance between the umbilicus and the pubic symphysis. **Conclusions:** Knowledge of anatomical landmarks can aid the surgeon in more efficiently harvesting the DIEP flap. Surface landmarks along the abdominal midline coupled with normalizing ratios can aid surgeons in predicting the location of the DIEA and its first perforator. The DIEA crosses the rectus at approximately two thirds of the distance between the umbilicus and pubis, and the first perforator can reliably be located at one half of this distance.

Breast reconstruction techniques have focused increasingly on using autologous abdominal wall tissue for several decades.^1^,^2^ Traditionally, this has been achieved with TRAM flaps. More recently however, emphasis has been placed on using adipocutaneous flaps and employing muscle-sparing techniques.^3^,^4^ The impact of these techniques has demonstrated fewer abdominal wall complications such as hernias, bulges, weakness, and shorter hospital stay.^5^

These efforts have culminated in identifying and employing the deep inferior epigastric artery (DIEA) and its perforators as an ideal vascular pedicle for free flap reconstruction. As the branching pattern and location of the DIEA vary among patients, the reconstructive surgeon is challenged to employ techniques to more effectively identify appropriate vessels for flap basis.

Understanding perforator characteristics is necessary to determine an ideal vessel for flap basis. Moon and Taylor describe major branching patterns as type I (single trunk), type II (bifurcation), and type III (trifurcation), with types I and II being the most common.^6^ Caliber and course are also important components of vessel characteristics. The DIEA courses between the anterior rectus sheath and the rectus muscle and will usually travel through the rectus muscle for part of its course. Medial and lateral branches have been associated with type II and III patterns.^7^ The lateral branch is in close proximity to the motor nerves to the rectus muscle. Extensive dissection of these lateral branches can denervate the rectus muscle and defeat the purpose of this muscle-sparing flap. Similarly, perforators with significant intramuscular components can lead to extensive muscle dissection and increased abdominal wall morbidity.^4^,^8^,^9^ Optimal perforator identification is essential as evidence suggests that basing the flap on a single large caliber (>1 mm) perforator with minimal intramuscular course results in decreased tissue loss and abdominal wall complications.

Several perioperative techniques to facilitate ease of identification of these vessels include Doppler ultrasonography, computed tomographic angiography, and magnetic resonance angiography.^10^ However, selection of the perforator is possible only through operative abdominal wall dissection. While anatomic and radiographic studies have shown the course and nature of the DIEA, none have demonstrated the use of ratio landmarks to predict the location of the DIEA and its perforators. We report our measurement of superficial landmarks to identify where the DIEA enters the rectus abdominis muscle along a longitudinal axis, the location of first DIEA perforator, and the application of these measurements to harvesting the deep inferior epigastric perforator (DIEP) flap.

## METHODS

**Figure 1:**
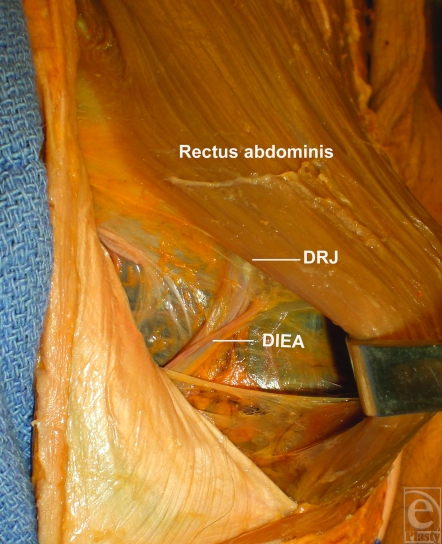
A dissection of the rectus muscle, the deep inferior epigastric artery (DIEA) rectus junction (DRJ), and the DIEA. Note the location of where the DIEA enters the rectus muscle.

**Figure 2:**
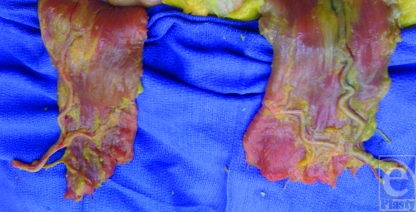
A dissection of the posterior aspect of the rectus muscle and the DIEA and its branching pattern.

**Figure 3:**
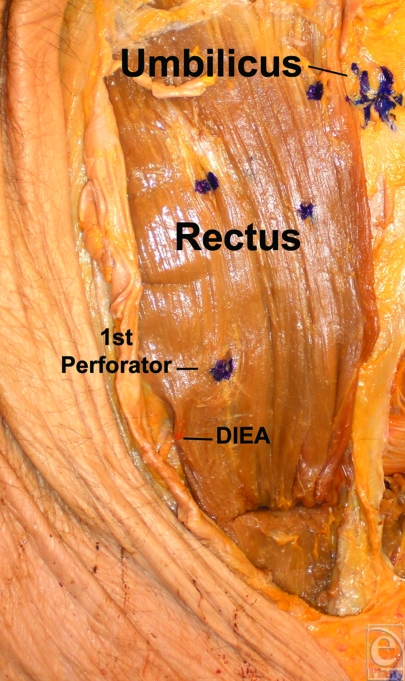
A dissection with the perforators marked.

Ten fresh cadaveric hemi-abdomens were dissected with the aid of loupe magnification. In this study, there were 4 male and 6 female specimens. Initial measurements were taken from the umbilicus to the xiphoid and the pubic symphysis. Through a midline abdominal approach, adipocutaneous flaps were elevated beyond the lateral border of the rectus muscle. The anterior rectus fascia was then incised along the lateral edge of the rectus muscle, and the DIEA was identified as it entered the lateral edge of the rectus muscle (Fig 1). Measurements were taken from the vertical height of this junction, the DIEA-RAM-junction (DRJ), to the umbilicus. Dissection proceeded with the elevation of the rectus muscle, the DIEA, and its perforators along the posterior aspect of the muscle (Fig 2). The first perforator of the DIEA was identified at the anterior rectus fascia layer and dissected down to its DIEA origin (Fig 3). Again, measurements were taken from the vertical height of the perforator to the umbilicus. In addition, transverse measurements were taken from the umbilicus to the lateral edge of the rectus and to the DIEA.

## RESULTS

The average distance from the xiphoid to umbilicus was 18.2 ± 1.27 cm. The distance from the umbilicus to pubis was 14.9 ± 2.3 cm. The vertical distance from the umbilicus to the DRJ was 10.45 ± 1.58 cm, and the vertical distance from the level of the umbilicus to where the first DIEA perforator traverses the RAM was 7.4 ± 1.64 cm (Table 1).

Distance ratios were then calculated for the DIEA and its first perforator. The distance between the umbilicus and the DRJ corresponds to approximately 0.7 times the distance between the umbilicus and the pubic symphysis. Similarly, the distance between the umbilicus and the first perforator corresponds to approximately 0.5 times the distance between the umbilicus and the pubic symphysis (Table 1). Additional analysis demonstrated consistent findings with regard to distance ratios for each specimen individually.

## DISCUSSION

The reconstructive surgeon is constantly challenged to investigate and develop techniques to improve functional and aesthetic outcome. Utilizing the DIEP flap for breast reconstruction provides appropriate aesthetic outcome while preserving abdominal wall function.^11^

The burden to the surgeon typically lies in planning out the flap harvest and elevating the flap with its pedicle. Bony landmarks of the xiphoid and pubis are easily identified. The midline and lateral edges of the rectus muscle and umbilicus can usually be palpated and inspected. We used these bony and soft tissue landmarks in conjunction with our findings to develop normalizing ratios to best determine where the DIEA crosses the rectus muscle and where the first perforator penetrates through the anterior surface of the rectus.

Variance in patient size can be corrected by using vertical distance ratios. For example, we noticed that female specimens had relatively shorter abdomens as represented by an average vertical height of 31.5 cm from the xiphoid to the pubis. On the other hand, male specimens have an average vertical height of 34.2 cm. Despite variations in patients' height, the individual distance ratios calculated for each specimen group were consistent with our overall distance ratios.

**Figure 4:**
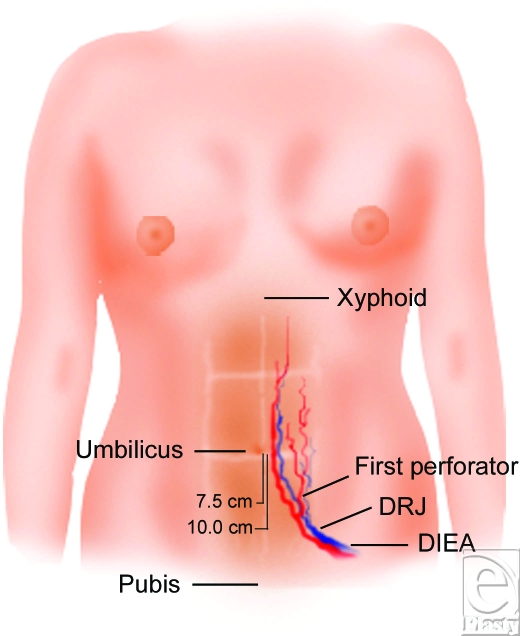
A schematic demonstrating the location of the deep inferior epigastric artery (DIEA), DIEA rectus junction (DRJ), and first perforator. Note the average distance from the umbilicus to the level of the first perforator and DRJ.

After measuring the umbilicus to pubis distance, the surgeon can utilize these ratios to predict the location of the DIEA and its first perforator. The DIEA crosses the rectus at approximately two thirds of the distance between the umbilicus and pubis and the first perforator can reliably be located at one half of this distance (Fig 4).

These distance ratios can also be used for harvesting a rectus muscle flap, a pedicled TRAM, or free TRAM flap. Traditionally, a fascial incision and extensive dissection along the lateral border of the muscle may be necessary to determine the location of the DIEA as it courses below the rectus muscle. The distance ratios described in this study can significantly enhance our ability to dissect and divide the flap inferiorly while avoiding injury to the DIEA system.

## CONCLUSION

Knowledge of anatomical landmarks can aid the surgeon in more efficiently harvesting the DIEP flap. We found that using longitudinal measurements between the umbilicus and the pubic symphysis along the abdominal midline can aid clinicians in predicting the location of the DIEA and its first perforator. These measurements are coupled with distance ratios to correct for variance in patient size. We anticipate that this study will decrease the need for extensive intramuscular dissection, associated morbidities of hernia, bulge, and denervation.

## Figures and Tables

**Table 1:** Anatomical measurements and ratios*

Cadaver	A, cm	B, cm	C, cm	D, cm	A/B	C/B	D/B	D/C
1	19.00	13.00	11.00	7.00	1.46	0.85	0.54	0.64
2	19.00	15.00	11.00	9.00	1.27	0.73	0.60	0.82
3	16.50	15.00	10.00	7.00	1.10	0.67	0.47	0.70
4	16.50	15.00	10.00	6.00	1.10	0.67	0.40	0.60
5	19.50	13.50	10.00	8.25	1.44	0.74	0.61	0.83
6	19.50	13.50	9.50	7.50	1.44	0.70	0.56	0.79
7	17.00	13.00	8.50	5.50	1.31	0.65	0.42	0.65
8	17.00	13.00	8.50	5.00	1.31	0.65	0.38	0.59
9	19.00	19.00	13.00	9.00	1.00	0.68	0.47	0.69
10	19.00	19.00	13.00	10.00	1.00	0.68	0.53	0.77
Mean	18.20	14.90	10.45	7.43	1.24	0.70	0.50	0.71
SD	1.27	2.32	1.59	1.64	0.18	0.06	0.08	0.08

*A indicates xiphoid—umbilicus; B, umbilicus—pubis; C, umbilicus—DIEA; D, umbilicus—first perforator.

## References

[1] Rozen W-M, Ashton M-W, Pan WR, Taylor GI (2007). Raising perforator flaps for breast reconstruction: the intramuscular anatomy of the DIEA. Plast Reconstr Surg.

[2] Nano M-T, Gill P-G, Kollias J, Bochner MA, Carter N, Winefield HR (2005). Qualitative assessment of breast reconstruction in a specialist breast unit. Aust N Z J Surg.

[3] Allen R-J (2003). DIEP versus TRAM for breast reconstruction. Plast Reconstr Surg.

[4] Chen M-C, Halvorson E-G, Disa JJ (2007). Immediate postoperative complications in DIEP versus free/muscle-sparing TRAM flaps. Plast Reconstr Surg.

[5] Kroll S-S, Sharma S, Koutz C (2001). Postoperative morphine requirements of free TRAM and DIEP flaps. Plast Reconstr Surg.

[6] Moon H-K, Taylor G-I (1988). The vascular anatomy of rectus abdominis musculocutaneous flaps based on the deep superior epigastric system. Plast Reconstr Surg.

[7] Rozen W-M, Palmer K-P, Suami H (2008). The DIEA branching pattern and its relationship to the perforators: the importance of preoperative computed tomographic angiography for DIEA perforator flaps. Plast Reconstr Surg.

[8] Blondeel N, Vanderstraeten G-G, Monstrey SJ (1997). The donor site morbidity of free DIEP flaps and free TRAM flaps for breast reconstruction. Br J Plast Surg.

[9] Futter C-M, Webster M-H, Monstrey SJ (2000). A retrospective comparison of the abdominal muscle strength following breast reconstruction with a free TRAM or DIEP flap. Br J Plast Surg.

[10] Rozen W-M, Stella D-L, Bowden J, Taylor GI, Ashton MW (2008). Advances in the pre-operative planning of deep inferior epigastric artery perforator flaps: magnetic resonance angiography. Microsurgery.

[11] Nahbedian M-Y, Momen B, Galdino G, Manson PN (2002). Breast reconstruction with the free TRAM or DIEP flap: patient selection. Plast Reconstr Surg.

